# HSP90 Inhibitor Ganetespib Enhances the Sensitivity of Mantle Cell Lymphoma to Bruton’s Tyrosine Kinase Inhibitor Ibrutinib

**DOI:** 10.3389/fphar.2022.864194

**Published:** 2022-06-03

**Authors:** Ziwen Lu, Zhixin Wang, Zhigang Tu, Hanqing Liu

**Affiliations:** ^1^ School of Pharmacy, Jiangsu University, Zhenjiang, China; ^2^ School of Life Sciences, Jiangsu University, Zhenjiang, China

**Keywords:** Hsp90, ganetespib, mantle cell lymphoma, Btk, ibrutinib, apoptosis

## Abstract

Mantle cell lymphoma (MCL) is a highly aggressive and heterogeneous B-cell lymphoma. Though Bruton’s tyrosine kinase (BTK) inhibitor ibrutinib has shown great efficacy as a single agent for MCL treatment, the real-world use of ibrutinib is still subject to limitations. Our previous study has shown the treatment with HSP90 inhibitor ganetespib can attack major targets of MCL, luckily complementary to ibrutinib’s targets. In this study, transient ganetespib treatment sensitizes MCL cells to ibrutinib as manifested by the significant decrease of IC_50_ values, percentages of EdU (5-Ethynyl-2′-deoxyuridine) positive cells, and levels of p-AKT and NF-κB after combinational treatment. Additionally, pretreatment with ganetespib enhanced cell cycle arrest induced by ibrutinib at G0/G1 phase and significantly decreased levels of cell cycle promoting proteins CDK2, 4, and 6. Pretreatment with ganetespib also enhanced cell apoptosis induced by ibrutinib through the upregulation of cleaved-caspase 9 and downregulation of BCL-2 in MCL cells at the molecular level. The sequential administration of ganetespib and ibrutinib had similar effects on increasing DNA damage as the transient treatment with ganetespib as demonstrated by the improved percentage of γH2AX and 53BP1 foci. Furthermore, ganetespib significantly increased inhibition of tumor growth mediated by ibrutinib *in vivo,* confirmed by the changes of the expression levels of Ki-67 and BCL-2 through immunohistochemistry assays. This study indicates that HSP90 inhibitor ganetespib maybe ideal for the combinational use with BTK inhibitor ibrutinib to target major pathogenesis-associated signaling pathways for MCL treatment which may help identify new possibilities for clinical trials.

## Introduction

Mantle cell lymphoma (MCL) is a highly lethal and aggressive B cell malignancy in the world (Jares and Campo, 2008; Sharma et al., 2010; Pérez-Galán et al., 2011; Jain and Wang, 2019; Yoon et al., 2020). Owing to the lack of effective therapeutic strategies, the median survival time of MCL is only about 4 years after initial diagnosis (Cheah et al., 2016). Therefore, a better understanding of the molecular pathogenesis is urgently needed to identify and design novel therapeutic strategies. MCL is characterized by cyclin D1 overexpression, hyper-activation of proliferation signals, defective DNA damage responses, deregulation of cell cycle progression, and resistance to apoptosis (Korz et al., 2002; Fernàndez et al., 2005). As a result, treatments targeting these disordered signaling pathways have achieved exciting success (Parekh et al., 2011; Zhao et al., 2017).

Ibrutinib is a selective and irreversible inhibitor of the Bruton’s tyrosine kinase (BTK). Since approved by FDA as a breakthrough therapy in 2013, the application of ibrutinib has changed the paradigm of the second-line therapy for MCL (Wang et al., 2013). To the best of our knowledge, ibrutinib is probably the most effective single agent for the treatment of MCL by far, but the real-world use of ibrutinib is still subject to limitations due to low rates of complete response, unacceptably high toxicity, and drug resistance (Wang et al., 2013; Cheah et al., 2015; Landau et al., 2017; Salem et al., 2019). Ibrutinib-based therapy is therefore actively explored and improved in combinational therapies (Kater and Brown, 2019). For example, clinical responses of ibrutinib was greatly enhanced in MCL when combined with BCL-2 antagonist venetoclax (Killock, 2018).

In our recent study (Liu et al., 2021), we found that transient treatment of MCL cells with the HSP90 inhibitor ganetespib can effectively inhibit the growth of MCL cells. Mechanistically, transient ganetespib treatment induced cell cycle arrest and significantly increased DNA damage accumulation in MCL cells.

Since ibrutinib can directly inhibit B cell receptor (BCR) signaling pathway and indirectly inhibit NF-κB and AKT pathways (Akinleye et al., 2013; Tanaka and Baba, 2020) and transient ganetespib treatment can induce cell cycle arrest and defects in DNA damage repair, we speculate that transient ganetespib treatment can synergize with ibrutinib by attacking the complementary major targets of MCL (Parekh et al., 2011). Moreover, because ganetespib is applied in a low dose for a short time and sequentially administered before the other targeted therapies, we believe such strategy may help increase the drug efficacy while preventing the accumulation of toxicities at the same time.

## Materials and Methods

### Cell Lines and Reagents

MCL cell lines Jeko-1 and Granta-519 were purchased from Cell Bank of the Chinese Academy of Sciences (Shanghai, China) and maintained in RPMI1640 medium supplemented with 10% fetal bovine serum (FBS) and penicillin/streptomycin (100 units/ml and 100 µg/ml, respectively) (all from Thermo Fisher Scientific). The cells were cultured at 37°C in a humidified, 5% CO_2_ atmosphere, and tested for *mycoplasma* contamination before experiments.

### Antibodies and Chemical Reagents

The commercial sources of antibodies used are listed in Supplementary Table S1. Ganetespib and ibrutinib were purchased from Selleck Chemicals, Inc. (Houston, TX). All chemicals were purchased from Sigma-Aldrich (Saint Louis, MO) unless otherwise specified. 10 mM stock solutions of ganetespib and ibrutinib were prepared in DMSO and stored at −20°C.

### Cell Viability

Jeko-1 and Granta-519 cells were plated in 24-well plates (5×10^4^ and 10×10^4^ cells/well, respectively), and treated with ganetespib or vehicle (DMSO, Sinopharm, Shanghai, China) for 72 h or briefly pretreated with ganetespib or vehicle for 12 h and ganetespib was then washed out. Then these cells were cultured in media with different concentrations of ibrutinib for various periods of time as indicated. Cell viability was determined using MTT (3- [4]-2, 5-diphenyltetrazolium bromide thiazolyl blue, Saiguo Biotech, Guangzhou, China). The cell viabilities were measured at least in three independent experiments. IC_50_ values were calculated using CompuSyn (ComboSyn, Inc., Paramus, NJ).

### Apoptosis and Cell Cycle Assays

Apoptosis and cell cycle assays were performed as previously described (Liu et al., 2015). Briefly, apoptosis was evaluated in drug-treated cells using Annexin V-FITC/propidium iodide (PI) staining kit (Yeasen, Shanghai, China). Jeko-1 and Granta-519 cells were trypsinized without EDTA and harvested. Cells were suspended in the binding buffer and incubated with Annexin V-FITC and PI staining reagents. For cell cycle assays, cells seeded in 6-well plates, harvested and stained with PI solution (KeyGen Biotech, Nanjing, China) with RNaseA (Biosharp, Hefei, China). Apoptosis and cell cycle samples were analyzed using a Beckman Coulter CytoFLEX Flow Cytometer (Brea, California). Data were analyzed using FlowJo software (FlowJo).

### EdU Staining

For EdU (5-Ethynyl-2′-deoxyuridine) staining, the harvested cells were stained with EdU (Ribobio, Guangzhou, Guangdong, China) after drug treatment, and then transferred to poly-lysine-treated adhesive slides, and then the slides were incubated and images of staining results were captured as previously described (Liu et al., 2021).

### Terminal Deoxynucleotidyl Transferase dUTP Nick End Labeling staining

Manufacturer’s protocol was followed. Briefly, the cells were fixed with 4% paraformaldehyde for 30 min and treated with 0.5% TritonX-100 for 7 min at room temperature. After washed once with PBS, the cells were incubated with Biotin-11-dUTP (KeyGen) for 30 min at 37°C in dark. Then the cells were incubated with streptavidin-fluorescein (KeyGen) staining solution for 30 min at 37°C in dark and stained with DAPI for 5 min (Roche, Mannheim, Baden-Württemberg, Germany). Images were captured using Nikon Eclipse (Tokyo, Japan) microscope.

### Immunoblot and Immunofluorescence

Immunoblot and immunofluorescence experiments were performed as previously described (Tu et al., 2011; Tu et al., 2013; Liu et al., 2015). In Immunoblot experiments, cells were lysed and protein concentrations were determined using BCA kits (Beyotime, Shanghai, China). Proteins were resolved on 8%-14% SDS-PAGE gels and transferred to PVDF membrane (Millipore, Billerica, MA). Membranes were blocked and then incubated at 4°C in primary antibody overnight, followed by incubation with HRP-conjugated secondary antibody and signal detection with SuperSignal West Pico Chemiluminescent Substrate (Thermo Fisher Scientific). In immunofluorescence experiments, Jeko-1 and Granta-519 cells were seeded on glass slides and treated as indicated. Then cells were fixed with freshly prepared 4% paraformaldehyde at R.T. for 10 min. Cells were incubated in 3% BSA/0.3% Triton X-100/PBS for 30 min, followed by 2 h incubation with primary antibodies in 3% BSA/0.3% Triton X-100/PBS at 22°C and incubation with appropriate secondary antibodies conjugated with Alexa Fluor 488 or Alexa Fluor 594 (Thermo Fisher Scientific) as indicated. Images were captured using Nikon Eclipse (Tokyo, Japan) microscope. The commercial sources of antibodies used are listed in Supplementary Table S1.

### *In Vivo* Experiments

All procedures involving mice were approved by the Institutional Animal Care and Use Committee of Jiangsu University. *In vivo* experiments were performed as previously described (Liu et al., 2021). All Nu/Nu nude mice were provided with sterilized food and water and housed in a barrier facility under a 12-h light/dark cycle. Six-week-old Nu/Nu nude mice were injected subcutaneously in the right flank with 2 × 10^6^ Jeko-1 cells. Tumors were measured every other day with vernier calipers, and tumor volumes were calculated using the equation: volume = 0.52 × W^2^ × L, where L and W represent the length and width of tumors, respectively. When tumor size reached approximately 100 mm^3^ (25 days after initial transplantation), the mice were randomized into four groups (six mice/group, containing vehicle+vehicle, ganetespib+vehicle, vehicle+ibrutinib, and ganetespib+ibrutinib groups). Ganetespib (15 mg/kg formulated in 10/18 DRD [10% dimethyl sulfoxide, 18% Cremophor RH 40, 3.6% dextrose, and 68.4% water] or 10/18 DRD [vehicle]) was administered once a week by tail vein injection for 10 days. Ibrutinib (50 mg/kg formulated in 10/18 DRD or 10/18 DRD [vehicle]) was administered once a day by intraperitoneal injection for 10 days. At the end of the observation, mice were sacrificed and autopsied. Tumors were removed and measured. Differences between two groups were compared by the Mann–Whitney test, with *p* < 0.05 considered significant.

### Tissue Preparation, Histology, and Immunohistochemistry

Tumors or tissues were paraffin embedded, sectioned (5 µm thick), and deparaffinized as previously described (Liu et al., 2021). Sections were either hematoxylin & eosin (H&E) stained or subjected to antigen retrieval for immunohistochemistry as previously described (Liu et al., 2021). Sections were exposed to appropriate species-specific secondary antibodies and exposed to 3,30-diaminobenzidine (KeyGen). Then sections were lightly counterstained with hematoxylin (KeyGen). The immunoreactivity scores were evaluated as previously described (Liu et al., 2015; Liu et al., 2021).

### Statistical Analysis

Data are presented as mean ± standard deviation from three independent experiments, unless otherwise stated. Statistically significant differences between different groups were determined by Student’s t tests (two-tailed) or one-way ANOVA followed by Bonferroni post-testing using GraphPad Prism version 5.00 (GraphPad, San Diego, CA) between different groups (^#^ *p* > 0.05; *p < 0.05; **p < 0.01; and ***p < 0.001).

## Results

### Both Ibrutinib and Ganetespib Suppressed Growth of MCL Cells *In Vitro*

**FIGURE 1:**
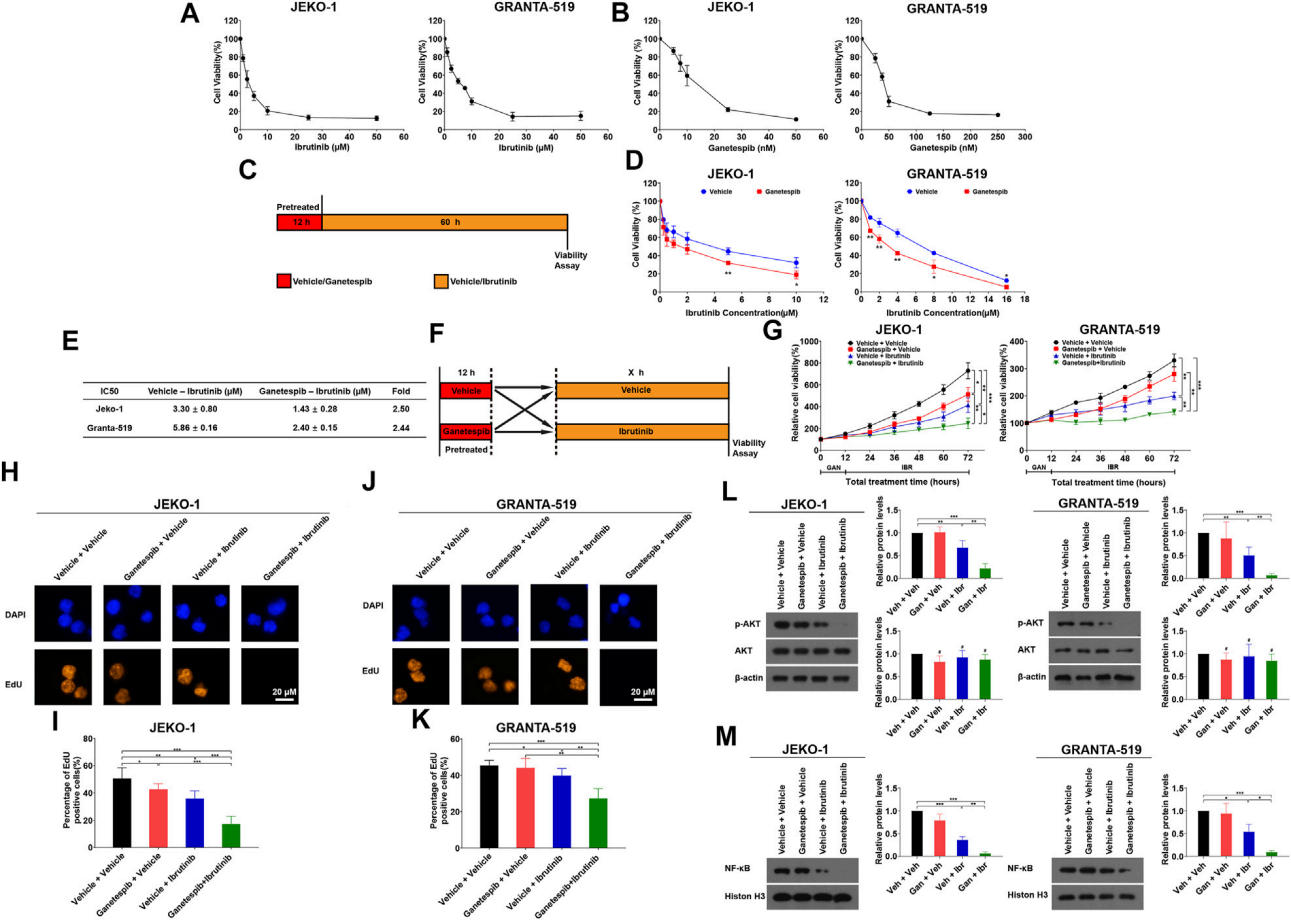
HSP90 inhibitor ganetespib enhanced inhibition of BTK inhibitor ibrutinib on proliferation of MCL cells. **(A)** Jeko-1 and Granta-519 cells were treated with ibrutinib at the indicated concentration in 0.5% DMSO for 72 h and the cell viability was assessed by MTT assays, respectively. **(B)** Jeko-1 and Granta-519 cells were treated with ganetespib at the indicated concentration in 0.5% DMSO for 72 h and the cell viability was assessed by MTT assays, respectively. **(C)** The scheme of experimental settings. Cells were pretreated with either ganetespib at the indicated concentrations or vehicle for 12 h and ganetespib was then washed out. Then the cells were treated with either different concentrations of ibrutinib or vehicle for 60 h before cell viability was assessed by MTT assays. **(D)** Jeko-1 and Granta-519 cells were treated with ganetespib (15 nM for Jeko-1 and 45 nM for Granta-519 cells, respectively) in 0.5% DMSO for 12 h, and then treated with different concentrations of ibrutinib as indicated in 0.5% DMSO for 60 h before cell viability was detected by MTT. **(E)** IC_50_ values calculated by using data from **(D)**. **(F)** The scheme of experimental settings. Cells were pretreated with either ganetespib (15 nM for Jeko-1 and 45 nM for Granta-519 cells, respectively) or vehicle for 12 h and then treated with either ibrutinib at 6 μM or vehicle for various periods of time as indicated before cell viability was assessed by MTT assays. **(G)**. Jeko-1 and Granta-519 cells were treated with either ibrutinib at 6 μM or vehicle for various periods of time as indicated before cell viability was assessed by MTT assays. **(H–M)** Jeko-1 and Granta-519 cells were pretreated with either ganetespib (15 nM for Jeko-1 and 45 nM for Granta-519 cells, respectively) or vehicle and then treated with either ibrutinib at 6 μM or vehicle for 24 h. **(H–K)** Representative pictures and the statistics of EdU staining of JEKO-1 **(H,I)** and Granta-519 cells **(J,K)**. **(L)** The protein levels and statistical analysis of p-AKT and AKT in Jeko-1 and Granta-519 cells treated as mentioned. **(M)** The protein levels and statistical analysis of NF-κB from nuclear extracts in Jeko-1 and Granta-519 cells treated as mentioned. Mean ± SD, *n* = 3.

At the beginning of our study, we tested the inhibitory effects of ibrutinib and ganetespib as single agents on Jeko-1 and Granta-519 cell lines. As shown in Figures 1A,B, the IC_50_ values of ibrutinib on Jeko-1 and Granta-519 cells were 3.24 ± 0.36 and 5.72 ± 0.40 μM, respectively. At the same time, the IC_50_ values of ganetespib on Jeko-1 and Granta-519 cells were 12.8 ± 1.57 and 45.7 ± 4.18 nM, respectively. These results demonstrated that both ibrutinib and ganetespib have potent inhibitory effects on cell proliferation of MCL cells.

### Transient Ganetespib Treatment Sensitized MCL Cells to Ibrutinib

In one of our recent studies, we found that transient exposure of ganetespib for only 12 h is sufficient to inhibit the growth of MCL cells (Liu et al., 2021). For the reasons previously mentioned, we hypothesized that pretreatment with ganetespib would probably increase the sensitivity of MCL cells to ibrutinib. In our experimental design, cells were pre-treated with ganetespib for 12 h, and then treated with different concentrations of ibrutinib for another 60 h, before the cell viabilities were measured (Figure 1C). As shown in Figure 1D,E, transient ganetespib treatment indeed increased the sensitivity of Jeko-1 and Granta-519 cells to ibrutinib as manifested by 2.50 and 2.44-fold decrease of IC_50_ values, respectively.

In the following experiments, MCL cells were pre-treated with ganetespib for 12 h, and then treated with ibrutinib for 12, 24, 36, 48, and 60 h, respectively (Figure 1F). Compared with the vehicle group and the single-agent groups, the combined use of the two drugs significantly inhibited cell proliferation of both Jeko-1 and Granta-519 cells, and the inhibitory effects of the combination therapy were significantly better than those of the single-agent groups (Figure 1G). Since DNA replication is an essential event of cell proliferation, EdU staining assays were also used to evaluate the effects of the combinational use of ganetespib and ibrutinib on cell proliferation of MCL cells. The concentrations of ganetespib and ibrutinib were set to the IC_50_ values determined earlier, and Jeko-1 and Granta-519 cells were pre-treated with ganetespib for 12 h, followed by ibrutinib treatment for another 24 h. Compared to the vehicle group, the combined use of the two drugs reduced the percentage of EdU positive cells from 50.09% to 16.42% in Jeko-1 cells, and also reduced the percentage of EdU positive cells from 45.39% to 27.36% in Granta-519 cells (Figures 1H–K). Additionally, the pre-treatment with ganetespib significantly reduced the percentage of EdU positive cells from 35.53% to 16.42% in Jeko-1 cells, and also reduced the percentage of EdU positive cells from 39.81% to 27.36% in Granta-519 cells when compared with ibrutinib single-agent group. Previous studies have found ibrutinib can inhibit NF-κB and AKT pathways, therefore the levels of these proteins were detected after treatment. Results demonstrated that ibrutinib reduced the protein levels of p-AKT in both cell lines, and the pretreatment with ganetespib led to larger reductions of the p-AKT levels (Figure 1L). Pretreatment with ganetespib also shows similar effects on NF-κB levels in nuclei in MCL cells (Figure 1M). These results supported that pretreatment with HSP90 inhibitor ganetespib sensitizes MCL cells to BTK inhibitor ibrutinib.

### Pretreatment With Ganetespib Enhanced Cell Cycle Arrest Induced by Ibrutinib at G0/G1 Phase

**FIGURE 2:**
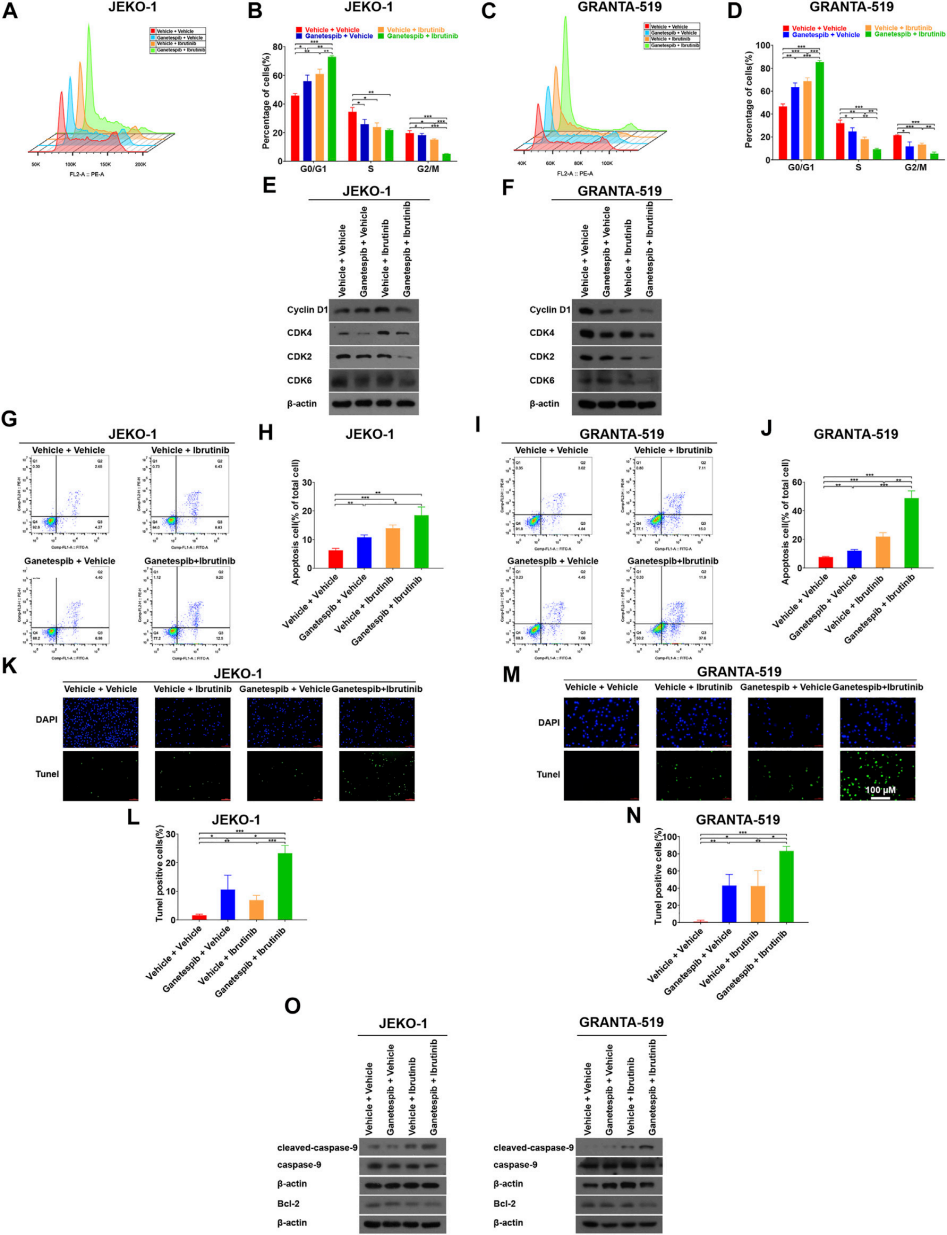
Pretreatment with ganetespib enhanced cell cycle arrest and cell apoptosis induced by ibrutinib. Jeko-1 and Granta-519 cells were treated with ganetespib (15 nM for Jeko-1 and 45 nM for Granta-519 cells, respectively) for 12 h, and then treated with either ibrutinib at 6 μM or vehicle for 12 h before cell cycle assays **(A–D)** or subjected to WB **(E–F)**. **(A,B)** Representative flow cytometry plots and statistics of cell cycle distribution for Jeko-1 cells. **(C–D)** Representative flow cytometry plots and statistics of cell cycle distribution for Granta-519 cells. **(E,F)**. Protein lysates of Jeko-1 **(E)** and Granta-519 cells **(F)** were subjected to immunoblot analysis with the indicated antibodies of cell-cycle markers. **(G–J)** Jeko-1 and Granta-519 cells were treated with ganetespib (15 nM for Jeko-1 and 45 nM for Granta-519 cells, respectively) for 12 h, and then treated with either ibrutinib at 6 μM or vehicle for 60 h before apoptosis assays. Representative flow cytometry plots and statistics of apoptosis distribution for Jeko-1 **(G,H)** and Granta-519 cells **(I,J)**. Cells were stained with annexin V–FITC/propidium iodide and analyzed for the presence of annexin V (+)/PI (-) (early apoptosis) and annexin V (+)/PI (+) (late apoptosis). **(K–O)** Jeko-1 and Granta-519 cells were treated with ganetespib (15 nM for Jeko-1 and 45 nM for Granta-519 cells, respectively) for 12 h, and then treated with either ibrutinib at 6 μM or vehicle for 12 h before TUNEL assays **(K–N)** or subjected to WB (**O**). Positive TUNEL cells show green fluorescence in combination with blue fluorescence DAPI (4′,6-diamidino-2-phenylindole) in the nuclei. **(K,L)** Representative pictures and statistics of TUNEL assays of Jeko-1 cells. **(M,N)** Representative pictures and statistics of TUNEL assays of Granta-519 cells. **(O)** The protein levels of apoptosis-associated markers cleaved caspase 9, caspase 9, and BCL-2 in Jeko-1 and Granta-519 cells treated as mentioned. Mean ± SD, *n* = 3.

To gain insight into the mechanism by which ganetespib pretreatment sensitizes MCL cells to ibrutinib, Jeko-1 and Granta-519 cells with different treatments were subjected cell cycle analysis. Consistent with our previous study (Liu et al., 2021), transient ganetespib treatment induced cell cycle arrest at G0/G1 phase. When ibrutinib was used as a single agent, it also induced G0/G1 cell cycle arrest. As expected, when the cells were treated with ganetespib and ibrutinib sequentially, the portions of G0/G1 phase increased significantly when compared to the vehicle or the single-agent groups in both cell lines (Figures 2A–D). Although MCL is characterized by the aberrant expression of cyclin D1 (Swerdlow et al., 2016), neither ganetespib nor ibrutinib showed obvious effects on cyclin D1 expression in either cell lines and there was not a consistent trend in the expression levels of Cyclin D1 after drug treatments in this study (Figures 2E,F). Instead, sequential administration of ganetespib and ibrutinib induced the decreases of the protein levels of CDK2, CDK4, and CDK6 dramatically in these two cell lines (Figures 2E,F). Because Lee et al. (2018) proposed that the activation of Myc can contribute to ibrutinib resistance, we examined the levels of c-myc after drug treatments and found that c-myc did not change significantly under our experimental conditions (data not shown).

### Pretreatment With Ganetespib Enhanced Cell Apoptosis Induced by Ibrutinib

Next, we studied the effects of sequential administration of ganetespib and ibrutinib on cell apoptosis. The results showed both ganetespib and ibrutinib induced mild but significant cell apoptosis in Jeko-1 and Granta-519 cells (Figures 2G–J). More importantly, ganetespib pretreatment could dramatically increase the levels of apoptosis (Figures 2G–J). In order to further confirm the above results, TUNEL assays were carried out in the following experiments. Consistently, the numbers of TUNEL-positive cells were significantly increased when cells were pretreated with ganetespib compared with the single-agent treated groups in Jeko-1 cells (Figure 2K,L). Similar results were obtained when Granta-519 cell were treated (Figures 2M,N). In addition, Western blot results showed the upregulation of cleaved-caspase9 and the downregulation of BCL-2 in MCL cells in ganetespib-ibrutinib combination group, which explained changes of cell apoptosis after drug treatment at the molecular level (Figure 2O).

### Effects of Combination Therapy on DNA Damage

**FIGURE 3:**
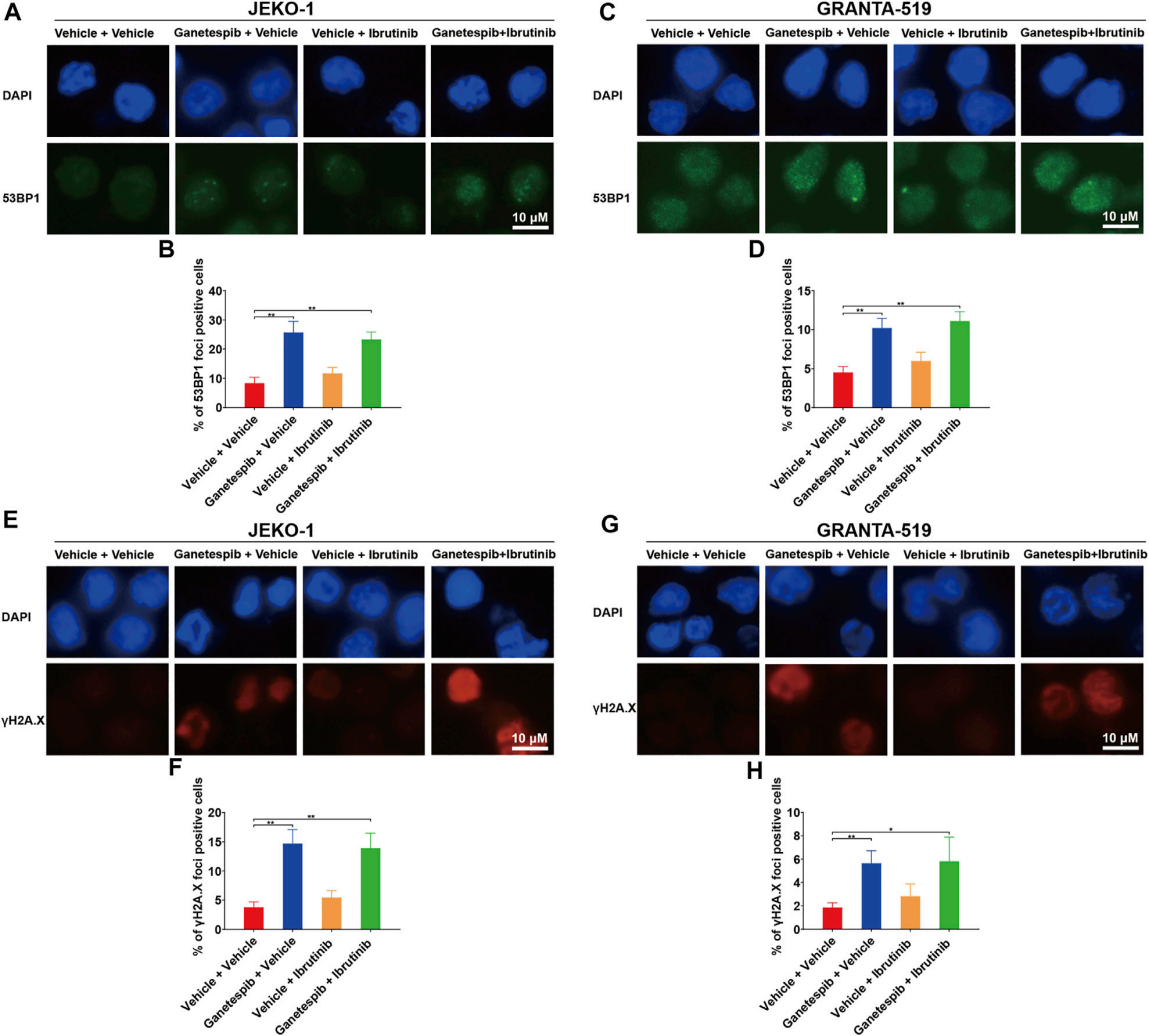
Effects of combination therapy on DNA damage. Jeko-1 and Granta-519 cells were treated with ganetespib (15 nM for Jeko-1 and 45 nM for Granta-519 cells, respectively) for 12 h, and then treated with either ibrutinib at 6 μM or vehicle for 60 h. **(A–D)** Representative pictures and statistics of immunofluorescence of 53BP1 in Jeko-1 **(A,B)** and Granta-519 cells **(C,D)**. **(E–H)** Representative pictures and statistics of immunofluorescence of γH2AX in Jeko-1 **(E,F)** and Granta-519 cells **(G,H)**. Mean ± SD, *n* = 3.

Our previous study found that transient ganetespib treatment resulted in increased DNA damage (Liu et al., 2021). Therefore, immunofluorescence (IF) analysis of γH2AX and 53BP1 foci were used to evaluate the effects on DNA damage by the drug treatment. Results showed that ganetespib treatment caused significant increase of numbers of positive 53BP1 foci in both cell lines as expected (Figures 3A–D). The combination use of ganetespib and ibrutinib also demonstrated similar effects on positive 53BP1 foci. Additionally, ganetespib treatment and the combination use of ganetespib and ibrutinib also increased the similar percentages of positive γH2AX cells (Figures 3E–H). These results demonstrated that the sequential administration of ganetespib and ibrutinib had similar effects on increasing DNA damage as the transient treatment with ganetespib.

### Ganetespib Increased Inhibition of Tumor Growth Mediated by Ibrutinib *In Vivo*

**FIGURE 4:**
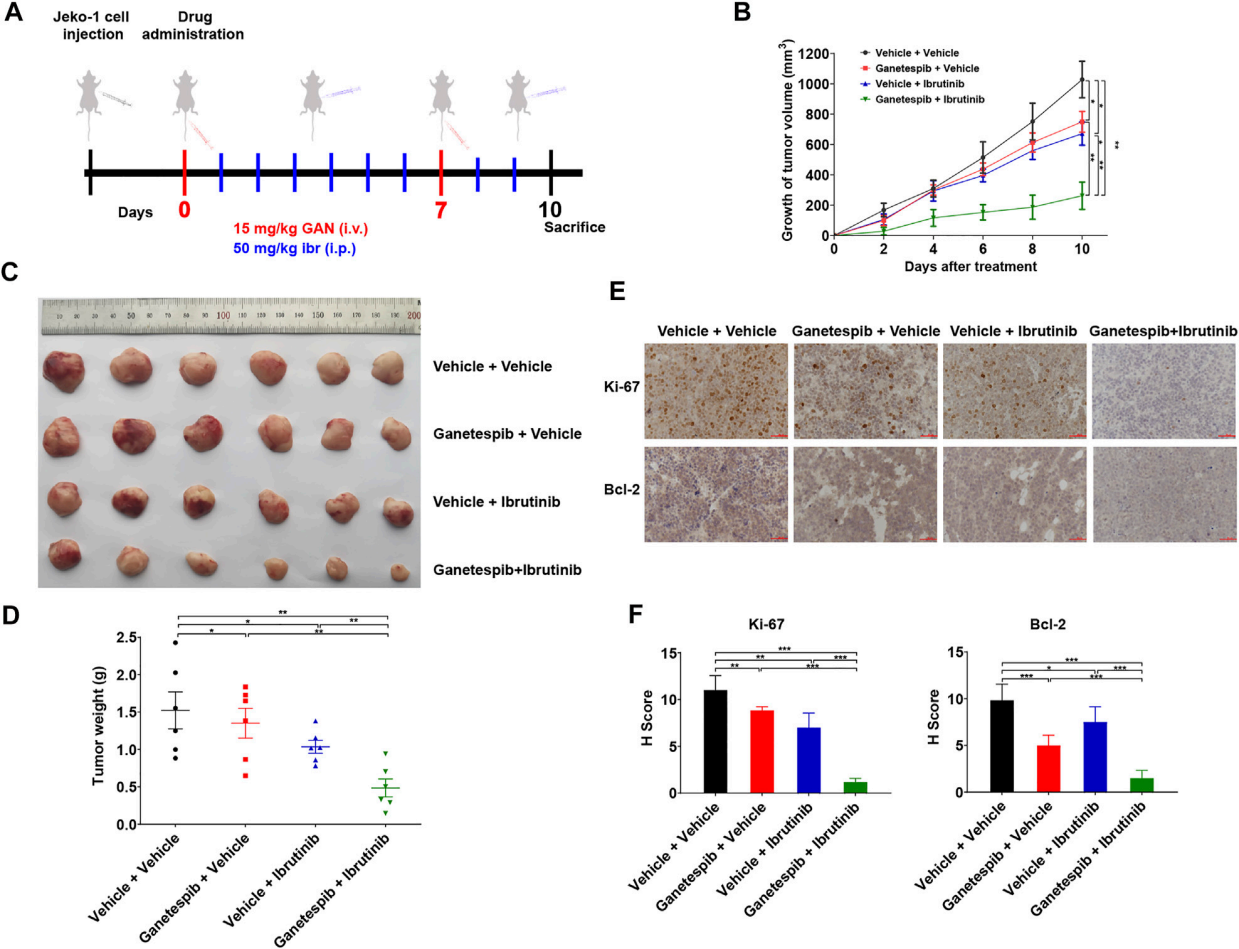
Ganetespib increased inhibition of tumor growth mediated by ibrutinib in the mouse model. **(A)**. The scheme of *in vivo* experimental settings. Mice bearing Jeko-1 xenografts (*n* = 6/group) were randomly grouped into four groups and treated with 15 mg/kg ganetespib (or vehicle) once weekly by i.v. and 50 mg/kg ibrutinib (or vehicle) daily by i.p. over a period of 10 days. **(B)**. Ganetespib significantly increased inhibition of tumor growth mediated by ibrutinib. Data show the average growth of tumor volumes calculated at the indicated days and the error bars are the S.D. **(C)**. The picture of the tumors at the day of sacrifice. **(D)**. Ganetespib significantly increased inhibition of tumor growth mediated by ibrutinib. Data show the average tumor weight and the error bars are the S.D. (*n* = 6/group). Differences among groups were compared by the Mann–Whitney test, with *p* < 0.05 considered significant (*p < 0.05; **p < 0.01). **(E,F)** Representative images of IHC staining and statistics of the expression of Ki-67 and BCL-2 in the tumors after treatment (scale bar = 50 μm). Mean ± SD, *n* = 3.

Finally, we evaluated the anti-tumor activity of ganetespib and ibrutinib on Jeko-1 xenograft tumors. When the tumor volumes reached about 100 mm^3^, these tumor-bearing mice were randomly grouped and treated as indicated in Figure 4A. As a matter of fact, the tumors in mice with single-agent treatment grew significantly slower than those of the vehicle group (Figures 4B,C). As expected, the combination therapy of ganetespib and ibrutinib dramatically inhibited the growth of xenografts, reducing final volume by 74.49% (*p* < 0.001), when compared to the vehicle group. More importantly, the ganetespib-ibrutinib treatment showed better tumor inhibitory effects than either ganetespib or ibrutinib single-agent groups. Consistent results were obtained in tumor weight after mice were sacrificed (Figure 4D). IHC results indicated that the expression levels of Ki-67 (a major proliferation biomarker of MCL) and BCL-2 were greatly reduced in tumors from mice treated with ganetespib-ibrutinib, while the extent of reduction in either ganetespib or ibrutinib groups were much less (Figures 4E,F). In addition, the weight measurement results of major organs suggested that all these drug treatment showed no obvious toxicities to mice (Supplementary Figure S1). Taken together, these data demonstrated that the combination of ganetespib and ibrutinib dramatically inhibited the growth of xenograft tumors without obvious toxicities.

## Discussion

Although the approval of ibrutinib has changed the treatment paradigm for recurrent MCL, the prognosis is still very poor even after the salvage therapy of ibrutinib (Cheah et al., 2015). Treatment failure of ibrutinib is usually due to inefficacy, toxicity, and acquired resistance (Wang et al., 2013; Mato et al., 2018; Rule et al., 2019). HSP90 inhibitors, such as ganetespib, are in a similar dilemma. So far, there are very few studies on the combination of HSP90 inhibitors and ibrutinib. Moreover, we planned to explore the roles of HSP90 inhibitors on further inhibiting BTK and its downstream pathways, such as NF-κB, in this study (Jacobson et al., 2016; Bergs et al., 2021).

According to the principles for designing combination therapy, the combination of ganetespib and ibrutinib was tried to target major pathogenesis-associated signaling pathways in this study, and achieved promising results *in vitro* and *in vivo*. In this study, transient ganetespib treatment resulted in cell cycle arrest and increased DNA damage, while ibrutinib inhibited BTK activity and the downstream AKT and NF-κB pathways. The combination of the two drugs induced more significant cell cycle arrest. In addition to the expected effects from transient ganetespib treatment, such as inducing cell cycle arrest and increasing DNA damage, we found that the combination of ibrutinib with transient ganetespib treatment led to some unexpected effects, such as the reduction of CDK2, 4, and 6 levels. The decreased levels of these cell cycle promoting proteins explains the more obvious blocking effects of the combined administration. Results from a recent clinical trial demonstrated that the combination of CDK4 inhibitor palbociclib and ibrutinib showed synergistic effects in patients (Martin et al., 2019). Such data have greatly increased our confidence in the future application of the combination of ganetespib and ibrutinib clinically.

Another clinical challenge for ibrutinib is that it often causes cells quiescent rather than killing them (Kater and Brown, 2019). In this study, we were excited to discover that the sequential treatment with ganetespib and ibrutinib significantly increased the proportions of apoptotic cells, which may be attributed to the significant reduction of the anti-apoptotic protein, BCL-2, by the combination of the two drugs. BCL-2 is known to be a good target for MCL (Davids, 2017; Khan and Kahl, 2018), and the clinical outcomes also showed that combined treatment using ibrutinib and BCL-2 antagonist venetoclax enhanced clinical responses (Pascual et al., 1998; Killock, 2018).

As for why CDKs and BCL-2 did not change significantly in single-agent treatment (either ibrutinib or transient ganetespib treatment), but changed significantly in combination, we can only speculate based on the current literatures. It is known that CDK2, 4, 6, and BCL-2 are transcriptionally regulated by NF-κB (Catz and Johnson, 2001; Iwanaga et al., 2008; Liu et al., 2011; Heine et al., 2018) and ibrutinib can indirectly inhibit NF-κB pathway when ibrutinib inhibits BTK. But such indirect inhibitory effects were not significant when ibrutinib was used alone. On the other hand, CDK2, 4, 6, and BCL-2 are the client proteins of HSP90. Although transient treatment with ganetespib had no significant effects on these proteins, the overlying effects from the two drugs resulted in significant decreases in these proteins. The specific mechanism remains to be further studied in the near future.

## Data Availability

The original contributions presented in the study are included in the article/Supplementary Material, further inquiries can be directed to the corresponding authors.

## Abbreviations

BCL-2, B-cell CLL/lymphoma 2; BTK, Bruton’s tyrosine kinase; 53BP1, tumor protein p53 binding protein 1; CDK, cyclin-dependent kinase; DAPI, 4′,6-diamidino-2-phenylindole; DMSO, dimethyl sulfoxide; EdU, 5-ethynyl-20-deoxyuridine; FBS, fetal bovine serum; HSP90, heat shock protein 90; γH2AX, phospho-Ser139 on H2AX; IF, immunofluorescence; MCL, mantle cell lymphoma; NF-κB, nuclear factor Kappa B subunit 1; p-AKT, phosphorylated-Akt.
