# Supplementary material for: HSP90 Inhibitor Ganetespib Enhances the Sensitivity of Mantle Cell Lymphoma to Bruton’s Tyrosine Kinase Inhibitor Ibrutinib
Source: Front Pharmacol. 2022 Jun 3;13:864194. doi: 10.3389/fphar.2022.864194 (PMC9204102; doi:10.3389/fphar.2022.864194)
Supplement: Supplementary file 1 [file Table1.docx]

**Supplementary Table 1. Antibodies used in this study.**

| **Primary antibody** | | | | |
| --- | --- | --- | --- | --- |
| **Targeted protein** | **Catalog No.** | **Source** | **Company** | **Application** |
| p-AKT | 9271 | Rabbit | Cell Signaling | Western blot, 1:1000 |
| AKT | 2920 | Rabbit |  |  |
| Cyclin D1 | 2978 | Rabbit |  |  |
| Cleaved-caspase9 | 52873 | Rabbit |  |  |
| NF-κB | 3035 | Rabbit |  |  |
| CDK2 | sc-53219 | Mouse | Santa CruzBiotech |  |
| CDK4 | sc-23896 | Mouse |  |  |
| CDK6 | sc-7961 | Mouse |  |  |
| Caspase 9 | sc-56073 | Mouse |  |  |
| Ki-67 | sc-23900 | Mouse |  | Immunohistochemistry, 1:100 |
| Bcl-2 | RLM3041 | Mouse | Ruiyingbio | Western blot, 1:1000  Immunohistochemistry, 1:100 |
| 53BP1 | A300-272A | Rabbit | BETHYL Laboratories | Immunofluorescence, 1:1000 |
| γH2A.X | 05-636 | Rabbit | Millipore |  |
| Histon H3 | 05-928 | Rabbit |  | Western blot, 1:1000 |
| β-actin | A1978 | Mouse | Sigma-Aldrich | Western blot, 1:1000 |
| **Secondary antibody** | | | | |
| Mouse IgG(H+L)-HRP | 115-035-003 | Goat | Jackson | Western blot, 1:5000 |
| Rabbit IgG(H+L)-HRP | 315-035-005 | Goat | Jackson | Western blot, 1:5000 |
| MouseIgG(H+L)-HRP-AlexaFluor488 | A-11070 | Goat | Invitrogen | Immunofluorescence, 1:1000 |
| Rabbit IgG(H+L)-HRP-AlexaFluor594 | A-11072 | Goat | Invitrogen | Immunofluorescence, 1:1000 |
