# Supplementary figures and images for: HSP90 Inhibitor Ganetespib Enhances the Sensitivity of Mantle Cell Lymphoma to Bruton’s Tyrosine Kinase Inhibitor Ibrutinib
Source: Front Pharmacol. 2022 Jun 3;13:864194. doi: 10.3389/fphar.2022.864194 (PMC9204102; doi:10.3389/fphar.2022.864194)

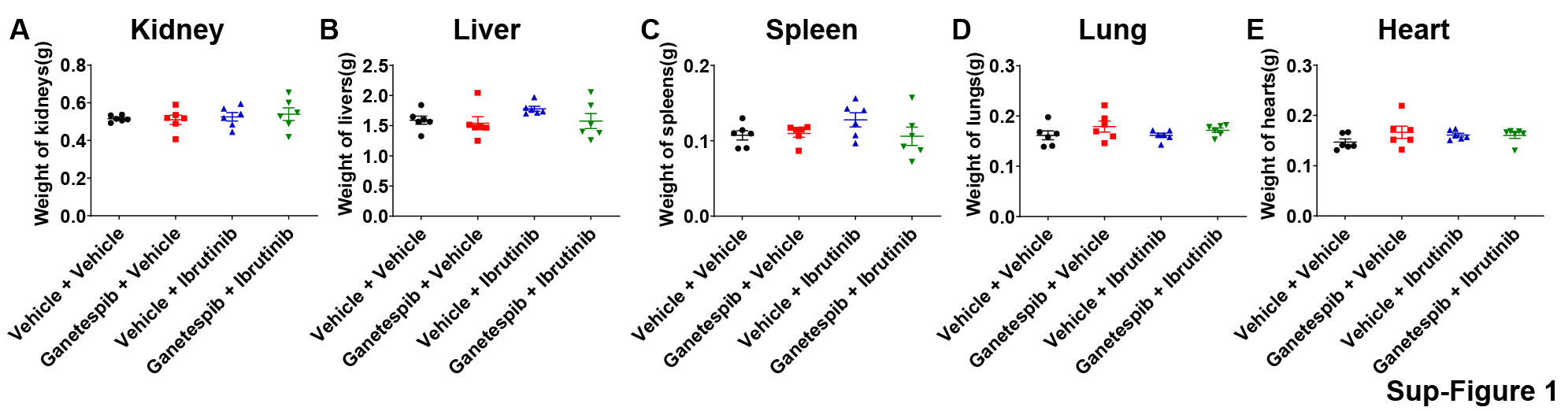

Supplement: Supplementary file 2 [file Image1.TIF]
