# Supplementary material for: HSP90 Inhibitor Ganetespib Enhances the Sensitivity of Mantle Cell Lymphoma to Bruton’s Tyrosine Kinase Inhibitor Ibrutinib
Source: Front Pharmacol. 2022 Jun 3;13:864194. doi: 10.3389/fphar.2022.864194 (PMC9204102; doi:10.3389/fphar.2022.864194)
Supplement: Supplementary file 3 [file DataSheet1.DOCX]

**Sup Figure 1. Weight of major organs after treatment with ganetespib and ibrutinib in the mouse model.** Statistics of weights of kidney (**A**), liver (**B**), spleen (**C**), lung (**D**), and heart (**E**) in different treatment groups.
